# The prognostic implications of SIRTs expression in breast cancer: a systematic review and meta-analysis

**DOI:** 10.1007/s12672-022-00529-7

**Published:** 2022-08-05

**Authors:** Hongchen Zhang, Chenyang Ma, Mingying Peng, Xiaoai Lv, Xiaohong Xie, Run Huang

**Affiliations:** 1 Department of Breast Surgery, The First Affiliated Hospital of Zhejiang Chinese Medical University, Hangzhou, 310000 China; 2 Department of Internal Medicine of Traditional Chinese Medicine, The Second People’s Hospital of Xiaoshan District, Hangzhou, 310000 China; 3 The First Clinical Medical College of Zhejiang, Chinese Medical University, Hangzhou, 310000 China; 4 Department of Liver Surgery and Transplantation, Liver Cancer Institute, Zhongshan Hospital, Fudan University, Key Laboratory of Carcinogenesis and Cancer Invasion of Ministry of Education, Shanghai, 200000 China

**Keywords:** SIRTs, Breast cancer, Prognosis, Meta-analysis

## Abstract

**Background:**

Sirtuins (SIRTs) have key roles in cancer progression. However, the prognostic implications of SIRTs in breast cancer (BC) remains a subject of debate and controversy. Thus, we performed a meta-analysis to identify the precise prognostic value of SIRTs in BC patients.

**Methods:**

Systematic literature searching was conducted in PubMed, Cochrane Library, Web of Science, and Embase databases. The pooled hazard ratios (HRs) with 95% confidence intervals (CIs) were calculated to estimate the association of SIRTs expression and survival outcomes in BC patients.

**Results:**

A total of 22 original studies with 6317 patients were eligible for this meta-analysis. The results showed that in patients with BC, elevated SIRTs levels were associated with shorter overall survival (OS) and disease-free survival (DFS) both in univariate (HR = 1.56, 95% CI 1.21–2.00; HR = 1.67, 95% CI 1.32–2.12, respectively) and multivariate analysis models (HR = 2.11, 95% CI 1.48–3.00; HR = 1.70, 95% CI 1.20–2.39, respectively). Notably, further subgroup analysis revealed that overexpression of SIRT1 and SIRT6 predicted poor OS (HR = 2.65, 95% CI 1.54–4.56; HR = 2.53, 95% CI 1.64–3.90, respectively) and DFS (HR = 1.65, 95% CI 1.07–2.56; HR = 2.74; 95% CI 1.88–4.01, respectively) in BC.

**Conclusions:**

Our data has elucidated that SIRT1 and SIRT6 could serve as prognostic biomarkers for patients with BC and may contribute to refined patient management.

**Supplementary Information:**

The online version contains supplementary material available at 10.1007/s12672-022-00529-7.

## Introduction

Breast cancer (BC), the most common malignancy worldwide, remains the pivotal cause of cancer-related mortalities for women [1]. On the molecular level, BC is categorized into four major subtypes: Luminal A and Luminal B (expressing the estrogen receptor), human epidermal growth factor receptor 2 (HER2) positive and triple-negative breast cancer (TNBC). Based on this molecular sub-classification, endocrine therapy, HER2-targeted therapy and chemotherapy have achieved considerable progress in clinical treatment of BC. Unfortunately, high incidence of recurrence and metastasis still resulted poor outcomes [2]. Even with the same tumor-node-metastasis (TNM) stage, the survival outcomes can vary dramatically. Clinically, to identify patients who are likely to have a poor prognosis is one of the major challenges. Therefore, uncovering novel prognostic biomarkers is urgently needed to assist the prediction of cancer survival and to facilitate the identification of therapeutic targets.

Sirtuins (SIRTs), which share homology with the yeast silent information regulator 2 (Sir2) gene, are a family of highly conserved nicotinamide adenine dinucleotide (NAD^+^)-dependent enzymes [3]. To date, seven sirtuin proteins (SIRT1-7) have been identified in mammals, with diverse cellular localizations. SIRT1 and SIRT6 are mostly localized in nucleus. SIRT2 is primarily found in the cytoplasm. SIRT3, SIRT4 and SIRT5 are mitochondrial SIRTs, while SIRT7 is present in the nucleolus [4]. Additionally, given their different enzymatic activities, SIRTs have a great diversity of biological functions. SIRT1 is a key regulator of cellular metabolism, life extension, inflammation, and tumorigenesis [5]. SIRT2 regulates numerous biological processes consisting of metabolism, mitosis regulation, and cell differentiation [6]. SIRT3, SIRT4 and SIRT5 serve at crucial junctions in mitochondrial metabolism [7]. SIRT6 promotes genome stability and DNA repair [8]. SIRT7 is a nuclear compartment involved in ribosomal biogenesis, senescence, and cellular stress responses [9].

Accumulating evidence has investigated that SIRTs expression were strongly associated with survivals and could function as independent prognostic biomarkers for cancers such as colorectal cancer [10], gastric cancer [11] and hepatocellular carcinoma [12]. However, due to the small sample sizes and insignificant or opposite results among several reports [13–15], no definite conclusion has been drawn in BC. By integrating all available evidence from related literature, meta-analysis could enhance the statistical power to obtain relatively accurate estimation. In the present work, we conducted a comprehensive meta-analysis and subgroup analysis to elucidate the prognostic value of SIRTs in BC patients.

## Methods

### Search strategy

Cochrane Library, Web of Science, Embase, and PubMed were comprehensively searched until the end of September, 2021. The search was limited to original articles published in English and as a full-text manuscript. We implemented the following search terms: (“breast cancer” OR “breast carcinomas” OR "breast neoplasm”) AND (“prognosis” or “survival” or “outcome”) AND (“SIRT1” OR “sirtuin 1” OR “SIRT2” OR “sirtuin 2” OR “SIRT3” OR “sirtuin 3” OR “SIRT4” OR “sirtuin 4” OR “SIRT5” OR “sirtuin 5” OR “SIRT6” OR “sirtuin 6” OR “SIRT7” OR “sirtuin 7”). All the references from the selected articles were furtherly scanned to identify more eligible studies.

### Inclusion and exclusion criteria

The selection of studies was completed independently by two authors (HZ and CM). The inclusion criteria were as follows: (a) having overall survival (OS), disease-free survival (DFS) or recurrence-free survival (RFS) outcomes; (b) patients were divided into a high expression group and a low expression group; (c) the diagnosis of BC was histologically and pathologically confirmed; (d) availability of sufficient data to calculate the hazard ratio (HR) and 95% confidence interval (CI); and (e) publication in English. The exclusion criteria were as follows: (a) duplicate or overlapping populations; (b) reviews, meta-analyses, case reports, letters, animal studies, and conference abstract; (c) studies without survival outcomes; and (d) studies published in a non-English language. Disagreements between the two reviewers were discussed to reach a consensus.

### Data extraction

Data extraction was done independently by two investigators (HZ and RH), including first author, year of publication, original nation, molecular subtype of BC, the studied SIRT type, sample size, antibody-related information, detection method, cut off value, survival outcome, HR with its 95% CI and analysis model. If a study did not report the HR, the survival information from Kaplan–Meier curves were digitized and extracted using Enguage Digitizer 4.1 [16]. Any conflicting results should be discussed with a third author (CM) until a consensus was reached.

### Quality assessment

Quality assessment was independently conducted by the same two researchers (HZ and RH) according to the Reporting Recommendations for Tumor Marker Prognostic Studies (REMARK) guidelines [17]. The REMARK checklist consist of 20 items and each item was answered to “adequate”, “inadequate” or “not evaluable” and given a score “2”, “1” or “0”. The highest score is 40. In case of disagreement, a consensus score was achieved after discussion.

### Statistical analyses

All the statistical analyses were done with the software STATA 16.0 (STATA Corporation, College Station, TX, USA). Heterogeneity among studies was determined using the chi-square-based Q and I^2^ test, with greater than 50% considered as high heterogeneity. A random-effects model was employed for analyses in the presence of substantial heterogeneity, otherwise, the fixed-effects model would be applied. Considering the almost identical definition, the DFS and RFS outcomes were merged in the meta-analysis. Subsequently, all the univariate and multivariate HRs were combined to evaluate the effect on prognosis respectively. To find the potential source of heterogeneity, the sensitivity analysis was performed. In addition, potential publication bias was detected using the funnel plot and Begg’s test. *P* value less than 0.05 was considered significant.

## Results

### Selection of studies

**Fig. 1:**
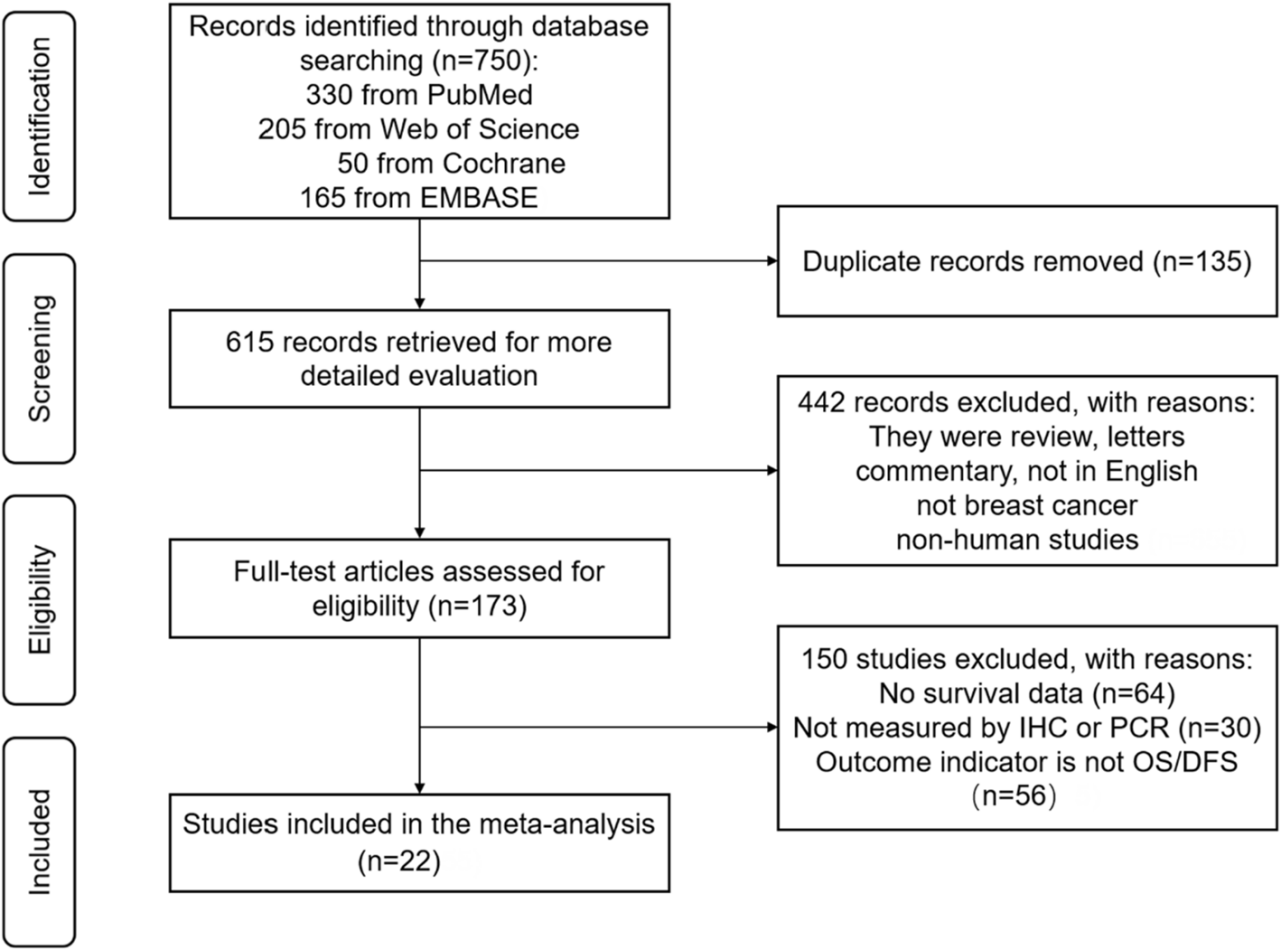
PRISMA diagram illustrating literature search and selection process

The initial search yielded a total of 750 potentially eligible records from the online databases. After removing duplicates, we screened 615 studies for further assessment. Then, 442 records were excluded based on studying the titles and abstracts after application of the exclusion criteria and 173 selected for full-text screening. Ultimately, 22 publications comprising 6317 samples were enrolled in this meta-analysis [18–39]. The PRISMA flow diagram for the study selection process is presented in Fig. 1.

### Characteristics of included studies

The 22 eligible articles were published between 2011 and 2020, from 7 different countries and the sample sizes ranged from 48 to 688. The expression level of SIRTs was mainly measured by immunohistochemistry (IHC), while only 4 studies used quantitative real-time polymerase chain reaction (qRT-PCR) as the detection method [27, 31, 32, 34]. Of note, differences were noticed among the publications concerning the antibodies used and the cut-off values implemented. A total of 7 different types of SIRTs were included in the analysis, including 10 studies of SIRT1 [18–27], 1 study of SIRT2 [28], 4 studies of SIRT3 [29–32], 1 study of SIRT4 [33], 1 study of SIRT5 [34], 4 studies of SIRT6 [25, 35–37], and 2 studies of SIRT7 [38, 39]. As for the survival outcomes, OS data were reported in 21 studies [18–20, 23–39], and the DFS outcomes were reported in nine studies [18–23, 25, 29, 37]. In terms of analysis model, multivariate analysis was conducted in 13 studies [18–23, 27–29, 33, 35, 37, 38] while univariate analysis in 19 studies [18–20, 23–37, 39]. In 20 of 22 studies, HR and 95% CI were obtained directly from the original articles. However, the data in two studies were extrapolated from Kaplan–Meier survival curves [36, 38]. According to the REMARK guidelines, the scores of these studies ranged from 27 to 36, indicating that the studies were of high quality. The overall characteristics of the included studies and the specific information of the antibodies are displayed in Tables 1 and 2, respectively.

**Table 1:** Characteristics of the studies included in the meta-analysis

Authors	Year	Country	SIRTs	Molecular subtype	Total cases	Survival analysis	Survival outcome	Score
Lee [18]	2011	Korea	SIRT1	ALL	122	U, M	OS/DFS	33
Wu [19]	2012	China	SIRT1	ALL	134	U, M	OS/DFS	34
				TNBC	51			
Derr [20]	2014	Netherlands	SIRT1	ALL	460	U, M	OS/DFS	35
Chung [21]	2015	Korea	SIRT1	HRBC	274	M	DFS	30
Jin [22]	2015	Korea	SIRT1	TNBC	319	M	DFS	31
Chung [23]	2016	Korea	SIRT1	TNBC	344	U, M	OS/DFS	32
Zhang [24]	2016	China	SIRT1	ALL	149	U	OS	29
Lee [25]	2016	Korea	SIRT1	ALL	688	U	OS/DFS	32
Tan [26]	2018	China	SIRT1	ALL	268	U	OS	32
Zhou [27]	2020	China	SIRT1	ALL	155	U, M	OS	30
Shi [28]	2019	China	SIRT2	ALL	296	U, M	OS	36
He [29]	2014	China	SIRT3	ALL	308	U, M	OS/DFS	32
Desouki [30]	2014	USA	SIRT3	TNBC	186	U	OS	30
Mas [31]	2016	Spain	SIRT3	HRBC	96	U	OS	28
Uzelac [32]	2020	Serbia	SIRT3	HRBC	63	U	OS	32
				TNBC	48			
Shi [33]	2016	China	SIRT4	ALL	409	U, M	OS	34
Greene [34]	2019	USA	SIRT5	ALL	626	U	OS	27
				TNBC	153			
Khongkow [35]	2013	UK	SIRT6	ALL	118	U, M	OS	28
Thirumurthi [36]	2014	USA	SIRT6	ALL	126	U	OS	27
Lee [25]	2016	Korea	SIRT6	ALL	688	U	OS/DFS	32
Bae [37]	2016	Korea	SIRT6	ALL	142	U, M	OS/DFS	33
Geng [38]	2015	China	SIRT7	ALL	144	M	OS	30
Huo [39]	2020	China	SIRT7	HRBC	335	U	OS	29

*U* univariate analysis, *M* multivariate analysis, *TNBC* triple-negative breast cancer, *HRBC* hormone receptor positive breast cancer, *OS* overall survival, *DFS* disease-free survival

**Table 2:** The information of the antibodies used in the included studies

Study	Sample	Detection method	Antibody	Dilution	Cut-off
Lee [18]	TMA	IHC	SIRT1(clone H-300) Santa Cruz Bio	1:50	P ≥ 30%
Wu [19]	TMA	IHC	SIRT1(clone H-300) Santa Cruz Bio	1:50	IRS ≥ 4
Derr [20]	TMA	IHC	SIRT1(ab32441), Abcam	1:200	P ≥ 70%
Chung [21]	TMA	IHC	SIRT1(clone H-300) Santa Cruz Bio	1:50	P ≥ 10%
Jin [22]	TMA	IHC	SIRT1(clone H-300) Santa Cruz Bio	1:50	P ≥ 10%
Chung [23]	TMA	IHC	SIRT1(clone H-300) Santa Cruz Bio	1:50	P ≥ 10%
Zhang [24]	TMA	IHC	SIRT1(#9475) Cell Signaling	NA	IRS ≥ 4
Lee [25]	TMA	IHC	SIRT1, Abcam	1:50	IRS ≥ 9.32
Tan [26]	TMA	IHC	SIRT1(#8469) Cell Signaling	1:25	IRS ≥ 4
Zhou [27]	TMA	RT-PCR	NA	NA	Fold change ≥ 2
Shi [28]	FFPE	IHC	SIRT2, Abcam	1:100	IRS ≥ 3
He [29]	FFPE	IHC	SIRT3(sc-99143) Santa Cruz Bio	1:200	IRS ≥ 5
Desouki [30]	TMA	IHC	SIRT3, Cell Signaling	NA	P ≥ 1%
Mas [31]	FFPE	RT-PCR	NA	NA	Fold change ≥ 2
Uzelac [32]	FFPE	RT-PCR	NA	NA	Fold change ≥ 2
Shi [33]	TMA	IHC	SIRT4(ab105039), Abcam	1:150	IRS ≥ 4
Greene [34]	FFPE	RT-PCR	NA	NA	NA
Khongkow [35]	TMA	IHC	SIRT6(#2590) Cell Signaling	1:50	IRS ≥ 4
Thirumurthi [36]	TMA	IHC	SIRT6(#2590) Cell Signaling	1:50	IRS ≥ 4
Lee [25]	TMA	IHC	SIRT6, Cell Signaling	1:50	IRS ≥ 109.8
Bae [37]	TMA	IHC	SIRT6, Lifespan Biosciences	1:50	IRS ≥ 5
Geng [38]	FFPE	IHC	SIRT7, Proteintech Group	1:100	IRS ≥ 5
Huo [39]	FFPE	IHC	SIRT7, Affinity Biosciences	1:100	P ≥ 10%

*TMA* tissue microarray, *FFPE* formalin-fixed paraffin-embedded, *IHC* immunohistochemistry, *P* percentage of stained cells, *IRS* immunoreactivity score considering both percentage of positive cells and staining intensities, *RT-PCR* real-time polymerase chain reaction, *NA* not available

### Association between SIRTs expression and OS

**Fig. 2:**
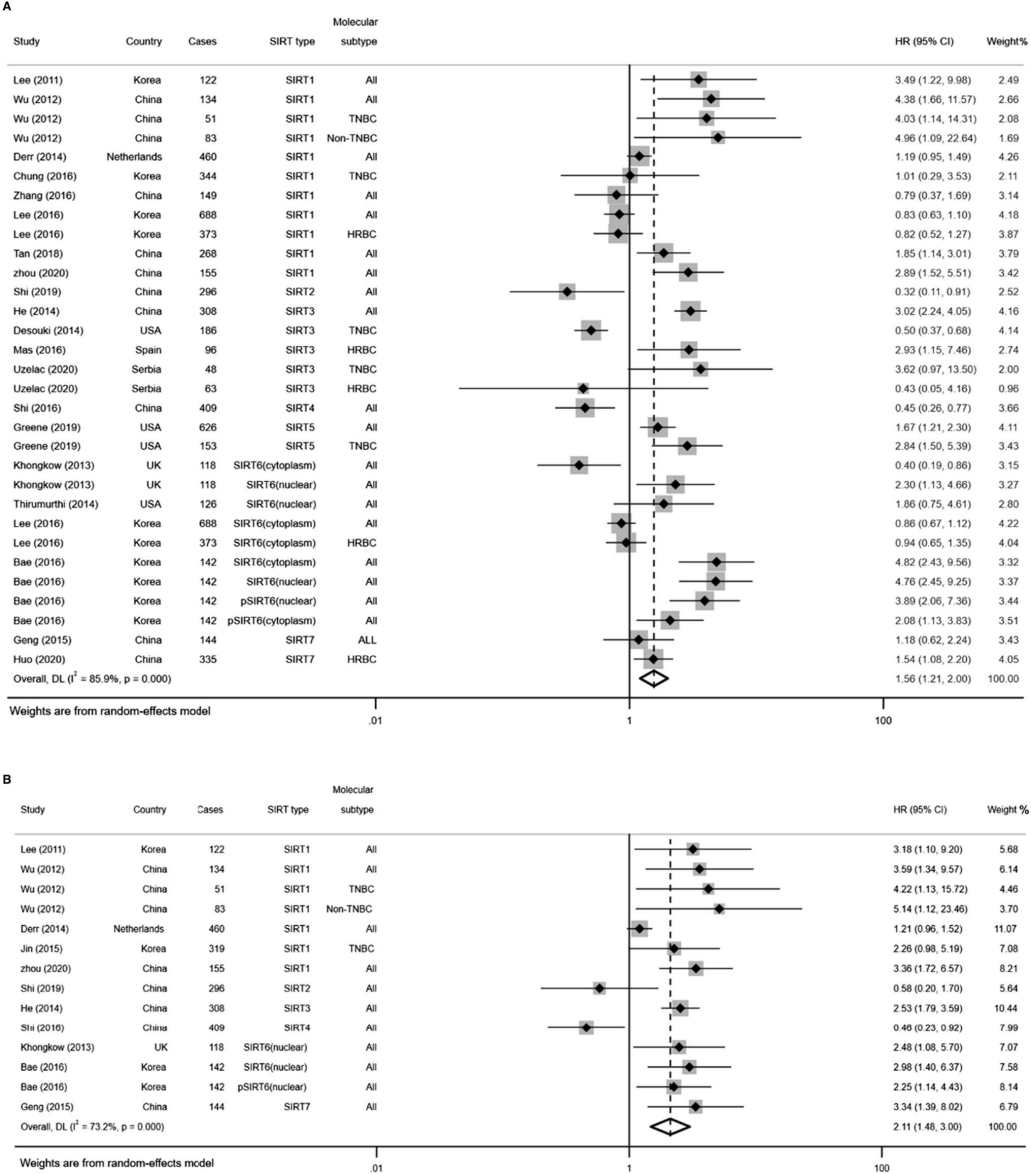
Forest plots for the association between high expression of SIRTs and OS with **A** univariate analysis and **B** multivariate analysis in BC

**Fig. 3:**
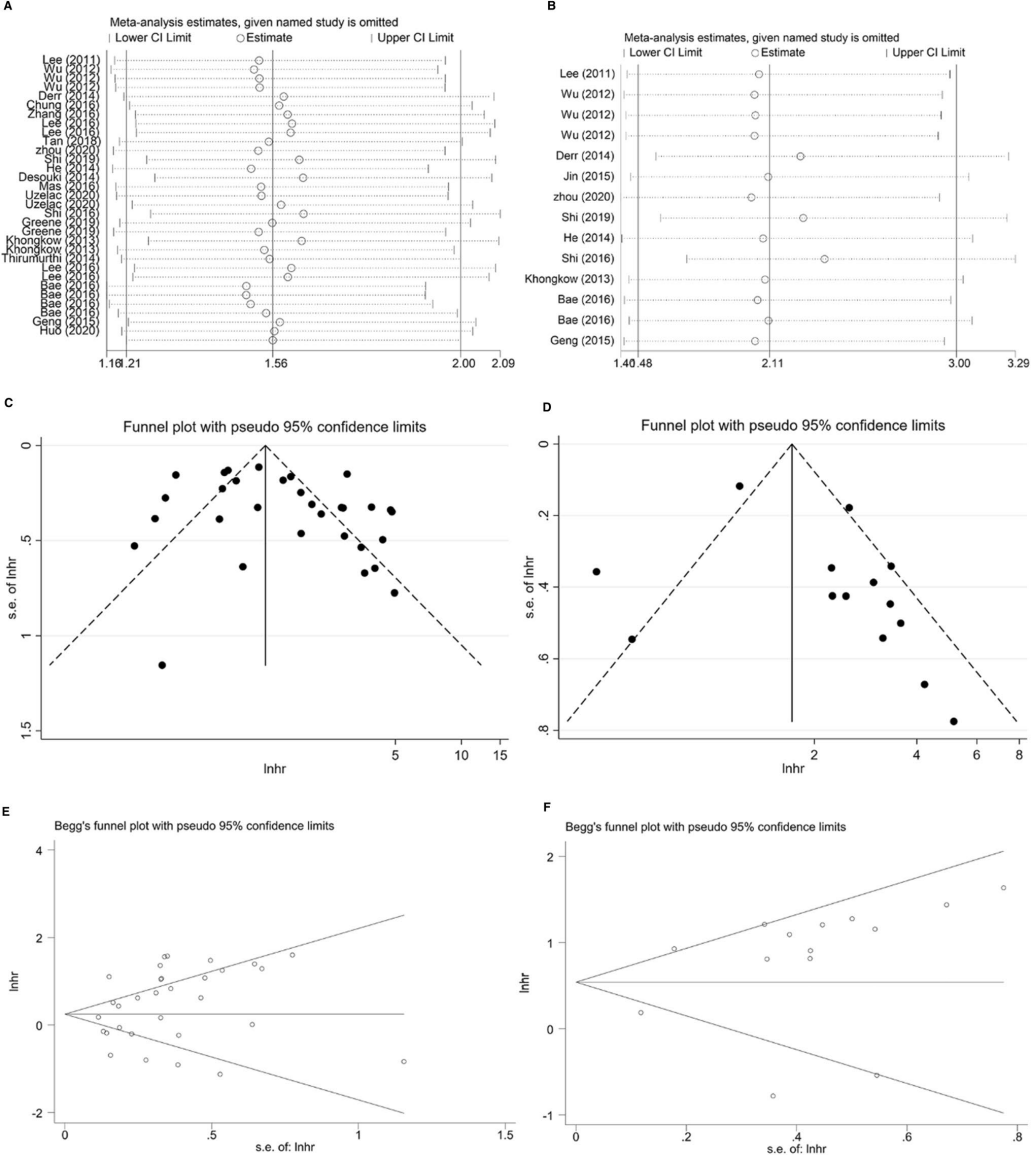
Sensitivity analyses of univariate analysis (**A**) and multivariate analysis (**B**) of OS; Funnel plots evaluating potential publication bias for OS in the univariate (**C**) and multivariate (**D**) analyses; Begg’s funnel plots for publication bias test in the univariate (**E**) and multivariate (**F**) analyses

The association between SIRTs expression and OS in BC was evaluated in 20 studies in a total of 5217 patients [18–20, 23–39]. In the presence of remarkable heterogeneity in both univariate (*I*^2^ = 85.9%, *p* = 0.000) and multivariate (*I*^2^ = 73.2%, *p* = 0.000) analysis models, the random-effects model was selected to compute the pooled HR and its 95% CI. Regardless of the SIRTs type, we found that higher SIRTs expression significantly linked with poor OS both in univariate and multivariate analyses (HR = 1.56, 95% CI 1.21–2.00; HR = 2.11, 95% CI 1.48–3.00; respectively; Fig. 2). Subsequently, sensitivity analysis was conducted to explore the sources of heterogeneity. Omitting each individual study did not influence the overall results significantly, sustaining the robustness of the merged results (Fig. 3A, B). Moreover, publication bias was assessed using the funnel plots and Begg’s test. As shown in Fig. 3, no obvious publication bias was found in the univariate (Begg’s test: *p* = 0.208) analysis. As for the multivariate model, a slight asymmetry in the funnel plot was shown to the right side of the pooled estimates (Fig. 3D), despite the non-statistically significant *p*-value of Begg’s test (0.913).

**Fig. 4:**
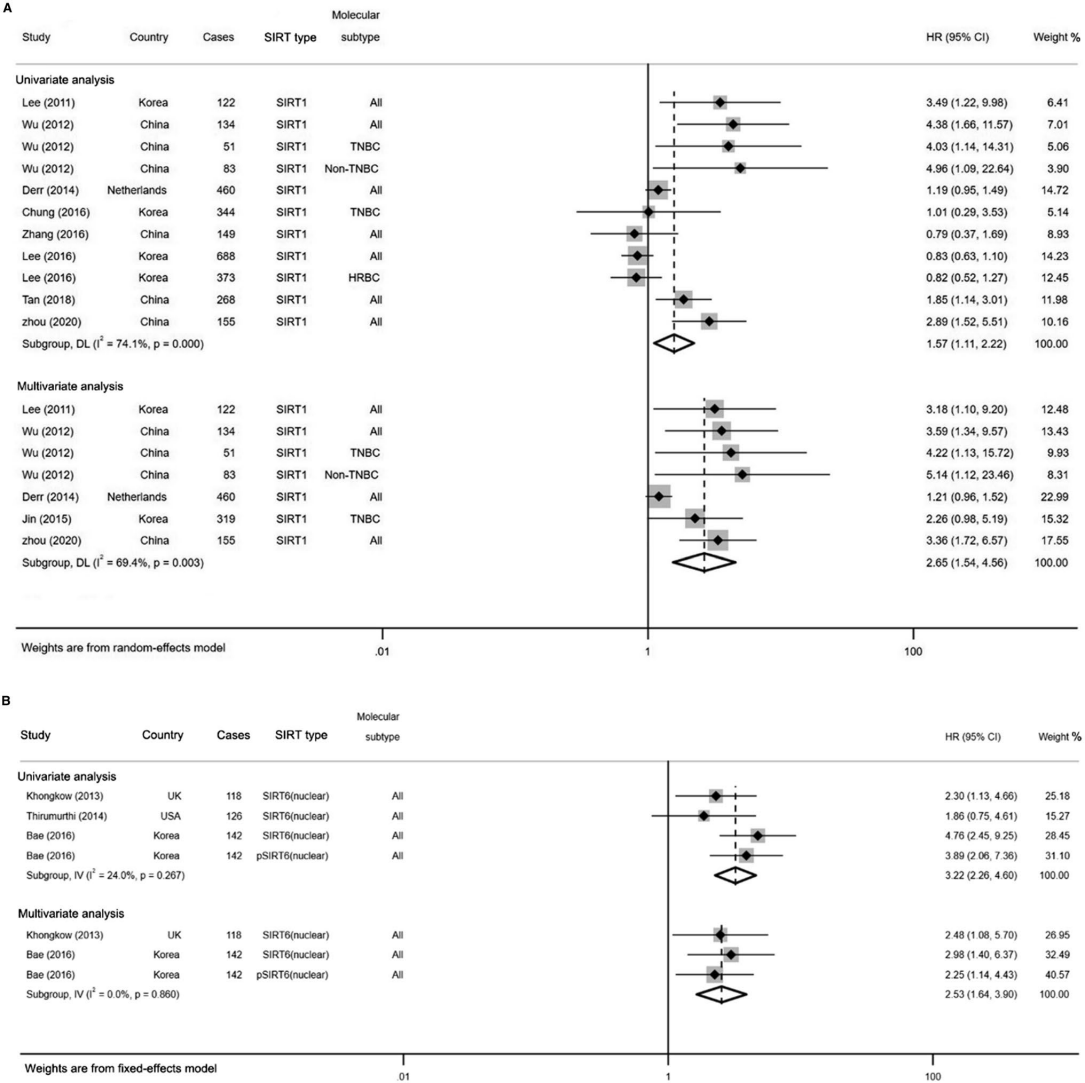
Forest plots for subgroup analysis of the association between SIRT1 (**A**), SIRT6 (**B**) overexpression and OS in BC

To explicate the heterogeneity, subgroup analysis was further conducted based on the type of SIRTs (Table 3). As shown in Fig. 4, both the univariate and multivariate analyses showed that high SIRT1 expression was significantly associated with poor OS (HR = 1.57, 95% CI 1.11–2.22; HR = 2.65, 95% CI 1.54–4.56; respectively) with moderate heterogeneity (*I*^2^ = 74.1%, *p* = 0.000; *I*^2^ = 69.4%, *p* = 0.003; respectively). Similarly, both the pooled results of univariate and multivariate estimates indicated a significant association between elevated SIRT6 (nuclear) expression and poor OS in patients with BC (HR = 3.22, 95% CI 2.26–4.60; HR = 2.53, 95% CI 1.64–3.90; respectively; Fig. 4B). The fixed-effects model was applied since there was no heterogeneity between studies (*I*^2^ = 24.0%, *p* = 0.267; *I*^2^ = 0.0%, *p* = 0.860; respectively). Additionally, stratified analysis by different molecular subtypes of BC revealed that elevated SIRT1 expression predicted a significantly worse OS in TNBC patients (HR = 2.70, 95% CI 1.34–5.45, *p* = 0.006, *I*^2^ = 0.0%) through multivariate analysis. However, no statistically correlation was noticed between SIRT3 expression and OS neither in TNBC (HR = 1.21, 95% CI 0.18–8.32, *p* = 0.848) nor in HRBC (HR = 1.50, 95% CI 0.25–8.97, *p* = 0.654) patients. Detailed results are shown in Table 4.

**Table 3:** Results of the subgroup analysis based on the type of SIRTs

SIRT	Endpoint	Univariate analysis				Multivariate analysis			
		HR	P	Heterogeneity		HR	P	Heterogeneity	HR
				*I*^2^ (%)	*P*			*I*^2^ (%)	*P*
SIRT1	OS	1.57(1.11, 2.22)	**0.001**	74.1	0.000	2.65(1.54, 4.56)	**0.000**	69.4	0.003
	DFS	1.54(1.05,2.24)	**0.026**	72.6	0.001	1.65(1.07,2.56)	**0.024**	76.7	0.000
SIRT3	OS	1.58(0.51,4.87)	0.423	94.7	0.000				
SIRT6 (cytoplasm)	OS	1.25(0.69,2.28)	0.466	87.8	0.000				
	DFS	1.40(0.92,2.12)	0.118	75.5	0.007				
SIRT6 (nuclear)	OS	3.22(2.26,4.60)	**0.000**	24.0	0.267	2.53(1.64,3.90)	**0.000**	0.0	0.860
	DFS	2.74(1.88,4.01)	**0.000**	0.0	0.439				

*OS* overall survival, *DFS* disease-free survival

The bold values indicate statistical significance, *P* < 0.05

**Table 4:** Results of the subgroup analysis based on the molecular subtype of BC

SIRT	Model	TNBC				HRBC			
		HR	P	Heterogeneity		HR	P	Heterogeneity	
				*I*^2^ (%)	*P*			*I*^2^ (%)	*P*
SIRT1(OS)	U	2.01 (0.52, 7.81)	0.313	57.0	0.127				
	M	2.70 (1.34, 5.45)	**0.006**	0.0	0.433				
SIRT1(DFS)	U					0.54(0.12,2.41)	0.423	78.9	0.03
	M	1.82 (1.28,2.59)	**0.001**	0.0	0.526				
SIRT3(OS)	U	1.21 (0.18,8.32)	0.848	87.9	0.004	1.50(0.25,8.97)	0.654	57.3	0.126

*U* univariate analysis, *M* multivariate analysis, *TNBC* triple-negative breast cancer, *HRBC* hormone receptor positive breast cancer, *OS* overall survival, *DFS* disease-free survival

The bold values indicate statistical significance, *P* < 0.05

### Association between SIRTs expression and DFS

**Fig. 5:**
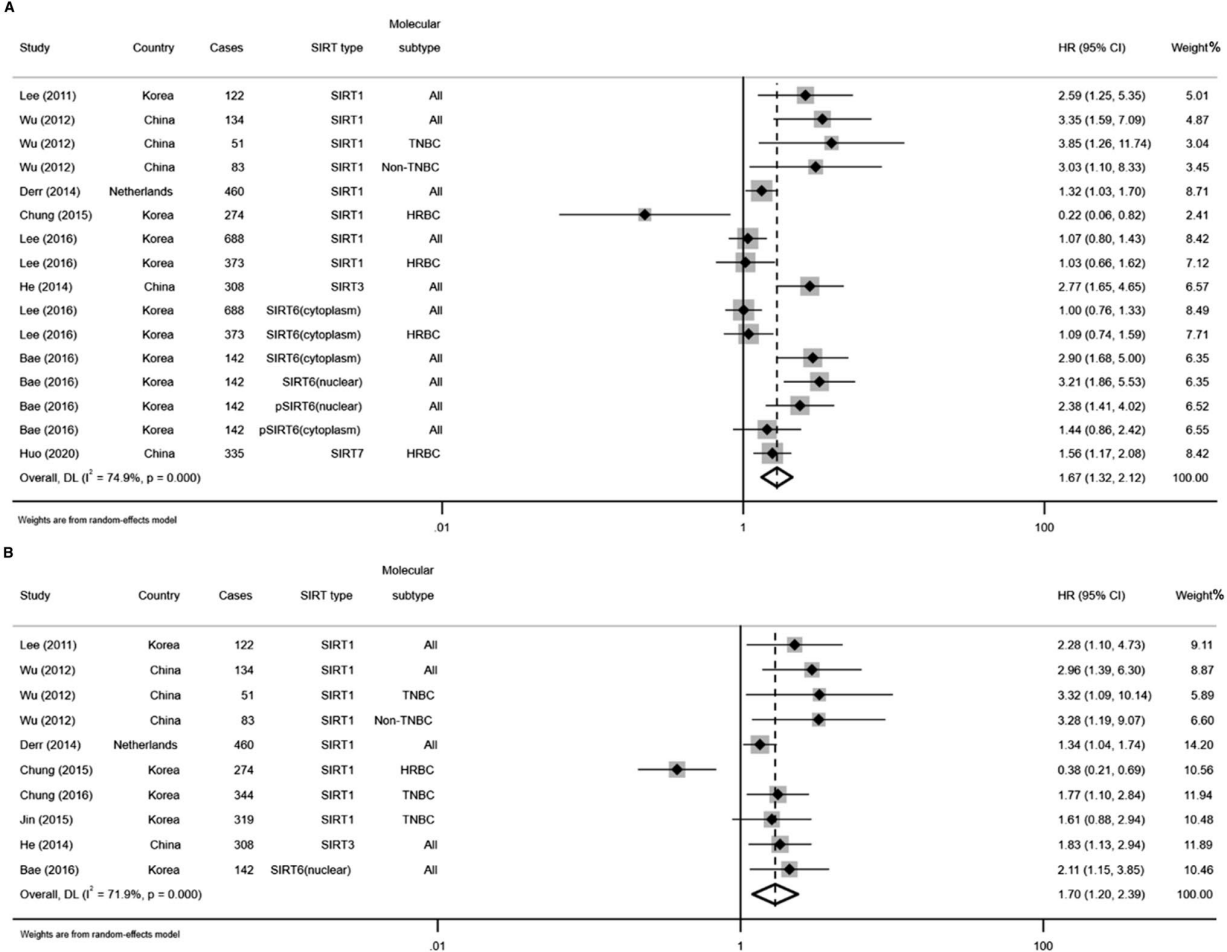
Forest plots of the association between high expression of SIRTs and DFS in patients with BC under different types of analysis. **A** univariate analysis; **B** multivariate analysis

**Fig. 6:**
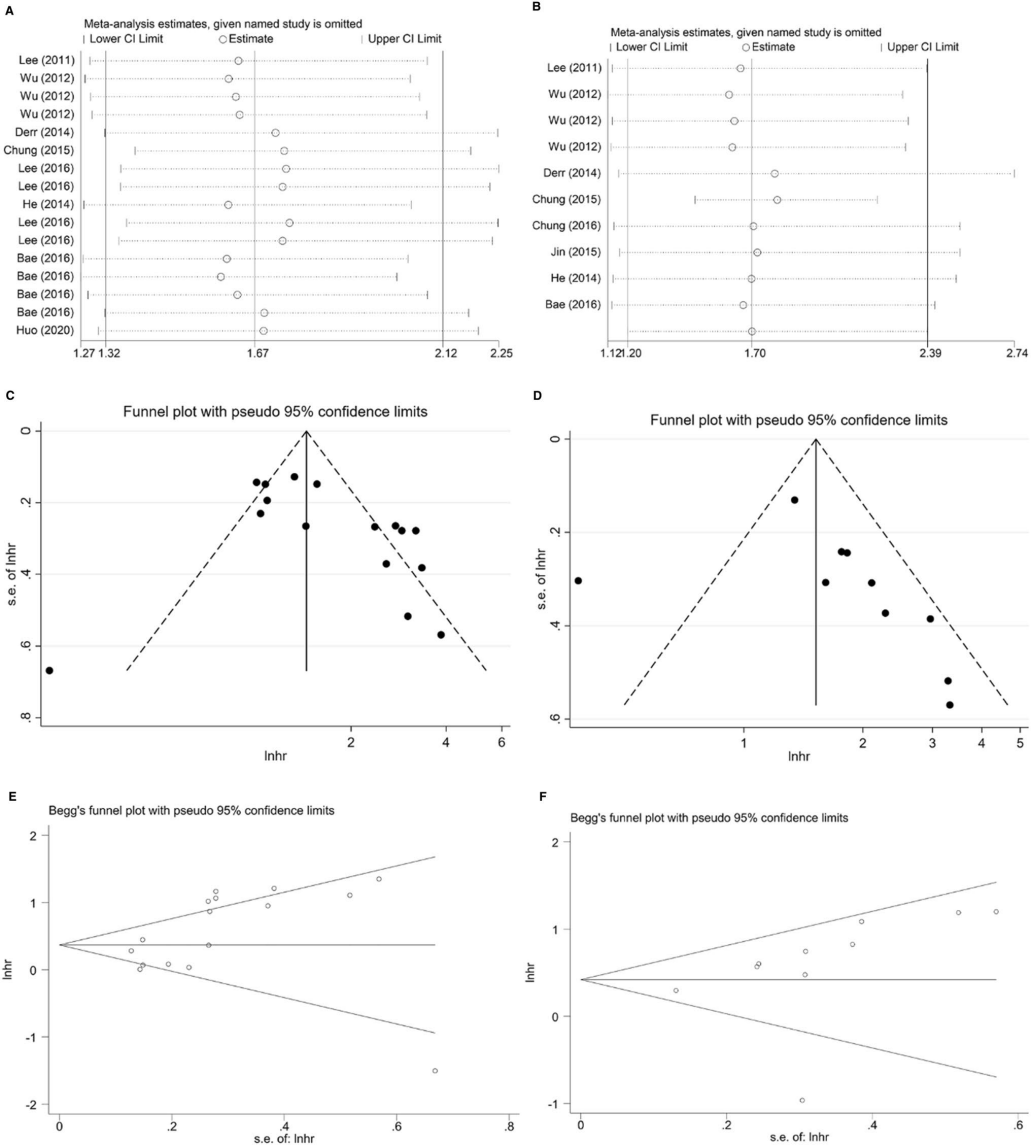
Sensitivity analyses of univariate analysis (**A**) and multivariate analysis (**B**) of DFS; Funnel plots evaluating potential publication bias for DFS in the univariate (**C**) and multivariate (**D**) analyses; Begg’s funnel plots for publication bias test in the univariate (**E**) and multivariate (**F**) analyses

A total of 9 studies evaluated the association between SIRTs expression and DFS in patients with BC [18–23, 25, 29, 37]. The random-effects model was used because of considerable heterogeneity existed in univariate analysis (*I*^2^ = 74.9%, *p* = 0.000) and multivariate analysis (*I*^2^ = 71.9%, *p* = 0.000). As shown in Fig. 5, the results were both statistically significant in pooling the univariate data (HR = 1.67, 95% CI 1.32–2.12) and multivariate data (HR = 1.70, 95% CI 1.20–2.39), indicating that patients with higher SIRTs expression had shorter DFS. The sensitivity analysis did not detect a study that could alter the combined results, which suggests that the results were reliable for DFS (Fig. 6A, B). Visual inspection of funnel plots indicated a potential publication bias for the univariate and multivariate analyses (Fig. 6C, D). Based on the statistical analysis, however, the Begg’s test (*p* = 0.063; *p* = 0.293; respectively) did not suggest the existence of obvious publication bias (Fig. 6E, F).

**Fig. 7:**
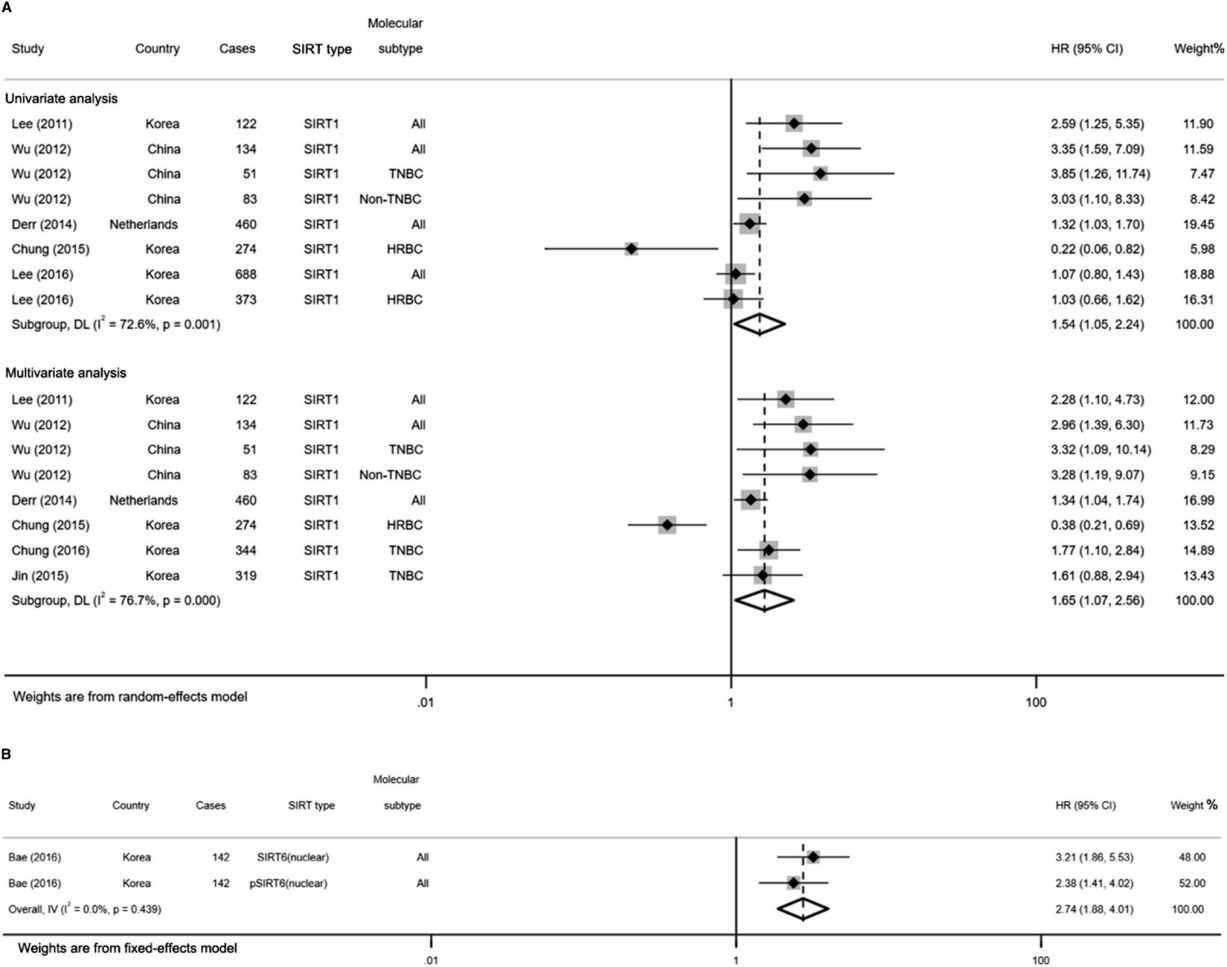
Forest plots for subgroup analysis of the association between SIRT1 (**A**), SIRT6 (**B**) overexpression and DFS in BC

Similar to OS analysis, the subgroup analysis was performed according to the type of SIRTs for DFS. As shown in Fig. 7, we found SIRT1 as a poor predictor of DFS both in the univariate (HR = 1.54, 95% CI 1.05–2.24, *I*^2^ = 72.6%, *p* = 0.001) and multivariate analysis (HR = 1.65, 95% CI 1.07–2.56, *I*^2^ = 76.7%, *p* = 0.000). Meanwhile, higher levels of SIRT6 (nulcear) expression were statistically associated with poorer DFS (HR = 2.74, 95% CI 1.88–4.01; *p* = 0.000, *I*^*2*^ = 0.0%) in fixed-effects model through univariate analysis (Fig. 7B). With regard to the molecular subtype of BC, the meta-analysis of multivariate estimates provided evidence that high SIRT1 expression was significantly related to poor DFS in TNBC (HR = 1.82, 95% CI 1.28–2.59) rather than in HRBC (HR = 0.54, 95% CI 0.12–2.41, *p* = 0.423; Table 3). The fixed-effects model was applied for low heterogeneity between the studies (*I*^2^ = 0.0%, *p* = 0.526).

## Discussion

The association between SIRT family members and survival outcomes of BC patients has been widely investigated, but no compatible results have been achieved. Numerous studies have discovered that SIRT1 overexpression was correlated with unfavorable OS in BC patients [18–20]. However, contradictory results were reported by Zhang et al. that patients with high SIRT1 expression had better OS [24]. He et al. reported that higher SIRT3 expression predicted inferior survival outcomes in BC [29], while opposite result was found by Desouki et al. [30]. Furthermore, most of the studies have demonstrated a worse survival outcome in association with a higher SIRT6 expression [35, 37], however, other studies presented insignificant or inverse results [25, 36]. These inconsistent results motivated us to perform this systematic review and meta-analysis to assess the prognostic value of different SIRTs in patients with BC. In the present work, we compiled and summarized the survival data of 6317 BC patients included in 22 eligible articles. The pooled data provided strong evidence that elevated SIRTs levels were significantly associated with shorter OS and DFS in both univariate and multivariate analyses. Regarding the type of SIRT, subgroup analysis further indicated that a shorter survival outcome was predicted by overexpression of SIRT1 and SIRT6 (nuclear). Therefore, SIRT1 and SIRT6 (nuclear) could serve as precise and available prognostic indicators and promising therapeutic targets for BC patients.

Histone deacetylases (HDACs) are major agents of epigenetic regulation and their dysfunctional deacetylase activity has been strictly related to the tumorigenesis process [40]. SIRTs (SIRT 1–7), which use NAD+ as a cofactor, are members of class III HDACs [41]. SIRT1 is widely recognized as a crucial epigenetic regulator implicated in many biological processes, including metabolism, genomic stability maintenance, aging, and tumorigenesis [42]. Acting as an established modulator, SIRT1 could induce the proliferation and invasion of BC cells via the deacetylation and subsequent degradation of important nuclear proteins, such as p53, E2F1, or NF-kB. By inhibiting p53 [43] or promoting the activity of PI3K/Akt signaling pathway [44], SIRT1 enhanced proliferation of BC cells. In nude mice models, miR-301 overexpression accelerates the progression of BC by mediating the SIRT1/SOX2 pathway [45]. Additionally, SIRT1 plays a pivotal role in the regulation of the epithelial-mesenchymal transition (EMT) process, which contributes to the metastasis of cancer cells. SIRT1 deacetylates and stabilizes the EMT inducer PRRX1, and promotes lung metastasis by upregulating KLF4 in BC cells and xenograft tumors [46]. SIRT1 could also upregulate matrix metalloproteinase-2 (MMP-2) level in BC cell lines by its deacetylation activity, exhibiting a direct correlation with advanced TNM stage, higher rates of lymph node metastasis (LNM) and poor survival of patients [47]. Notably, our subgroup analysis according to the molecular subtype showed that SIRT1 overexpression predicted a significantly worse OS in TNBC patients. Wu et al. asserted an oncogenic role of SIRT1 in TNBC subtype. They showed that SIRT1-mediated activation of AMPK selectively inhibits fibronectin-dependent migration of TNBC cells [19]. In vitro experiments, down-regulated expression of SIRT1 in TNBC cells inhibited tumor invasion, with altered expression of EMT-related proteins [22]. Moreover, SIRT1 knockdown in TNBC cells resulted in a decrease in hTERT expression, and increased cellular apoptosis [48]. Recent reports further demonstrated that BRCA1 could induce expression of endogenous SIRT1 in TNBC cells, revealing a novel molecular mechanism underlying TNBC EMT leading to lung metastasis in mouse model [49]. It should be noted that SIRT1 now becomes a potential target therapeutic site for BC. A wide range of chemical compounds that modulate SIRT1 activity were designed and tested, such as sirtinol, salermide, splitomicin and resveratrol [50]. A randomized clinical trial has documented that resveratrol had a dose-related impact on DNA methylation and prostaglandin E2 (PGE2) expression in women with high risk of BC. In the study, a decrease in methylation of the cancer-related gene RASSF-1α was observed. Meanwhile, proto-oncogene PGE2 was also found to be suppressed in the BC patients [51]. Another pilot clinical study of resveratrol in postmenopausal women concluded that daily 1 gm dose of resveratrol has favorable effects on estrogen metabolism, which is closely related to a higher incidence of BC. Their findings provided strong evidence for the preventative effect of resveratrol against BC [52]. However, the results need to be confirmed in future large scale prospective studies. Overall, clinical studies investigating the therapeutic potential of SIRT1 in cancer treatment hold promising results, proving the antitumor activity of SIRT1 modulators in BC.

Being a predominantly nuclear member of the SIRT family, SIRT6 has a pivotal role in DNA repair, genome maintenance and glucose metabolism [8]. Many studies assert an oncogenic role of SIRT6 in breast carcinogenesis. For instance, SIRT6 could enhance oxidative phosphorylation (OXPHOS), ATP/AMP ratio, and intracellular calcium concentration through its enzymatic activity. Meanwhile, compared to the Sirt6^+/+^ mice, in vivo dada showed that Sirt6 deletion repressed mammary tumor development and increased survival in Sirt6^+/−^ mice [53]. Nuclear factor-kappa B (NF-κB) is a key regulator of cancer metastasis, which can directly accelerate cell migration and invasion via the EMT process. SIRT6 upregulation could suppress the activation of NF-κB and the followed EMT process, resulting the impaired TNBC cell migration in vitro and in vivo [54]. Besides, SIRT6 overexpression has been shown to induce resistance to epirubicin and paclitaxel by activating DNA repair pathways [35]. Given the involvement of SIRT6 in BC progression, inhibition of SIRT6 may represent a successful strategy for cancer management. Sociali et al. developed a lysine-based compound targeting SIRT6 deacetylase and deacylase activities in the MCF-7 cell line. As a result, the activities of key glycolysis enzymes were increased and TNF-α secretion was reduced, in line with SIRT6 involvement in downregulation of glycolytic enzymes and the ability to trigger TNF-α secretion [55]. However, the potential therapeutic significance of SIRT6 in BC should be determined in the future.

SIRT3 is capable of metabolic reprogramming and contributes greatly in the fate of cancers. Up to date, tumor-suppressive and oncogenic roles of SIRT3 were both discussed in BC [56]. The most important tumor suppression role of SIRT3 is that it hinders cancer metabolism changes via the inhibition of hypoxia-inducible factor-1a (HIF1α) [57]. Moreover, Zhang et al. discovered a small-molecule activator of SIRT3 can inhibit the proliferation and migration of BC cells through SIRT3-driven autophagy/mitophagy signaling pathways [58]. In addition, SIRT3 could inhibit the oncogenic capacity of BC cells via increasing p53 expression and could suppress BC metastasis by repressing Src oxidation [59]. However, some studies hold the opposite view. It is reported that the activation of superoxide dismutase 2 (SOD2) mediated by SIRT3 can promote EMT in TNBC cells [60]. Meanwhile, He et al. revealed that SIRT3 overexpression was significantly correlated with clinical characteristics including LNM, pathological grade and tumor size of BC [29]. In this meta-analysis, no detectable relation was found between SIRT3 expression and prognosis in BC. Likewise, similar results were found in a certain molecular subtype of BC. Probably due to relatively fewer studies, the results remain inconclusive and require further comprehensive investigations. As for SIRT2, 4, 5 or 7, we did not perform subgroup analysis due to the lack of adequate estimates. Therefore, further studies with adequate sample size are still needed for these members of the SIRT family and the results of this proposed study should be updated to include future research.

Nevertheless, this analysis has several limitations. First, only studies published in English were included in our meta-analysis, which is likely to result in selection bias. A bias may also exist due to the positive results reported in most of the including publications, which may have led to overestimations in our analysis. Second, there was statistical heterogeneity in this analysis, which might be partially explained by different antibody sources, different cut-off values and different detection methods. Third, some HR values were extracted from Kaplan–Meier curves, which may partly compromise the precision of the data. Forth, a difference in protocols for treatment after surgery in various studies might have a great impact on survival outcomes and thus resulted in some heterogeneity. Finally, the small sample sized articles lacking statistical power may affect the reliability of the results.

In conclusion, our meta-analysis highlights that elevated SIRTs levels were significantly associated with shorter OS and DFS via combining univariate and multivariate analyses. Particularly, SIRT1 and SIRT6 could serve as prognostic biomarkers for patients with BC and may lead to refined patient management. However, these results should be validated in well-designed prospective cohort studies, and the clinical role of SIRTs for BC deserves further investigation.

## Supplementary Information

**Additional file 1.** Raw data.

## Data Availability

All data generated or analysed during this study are included in this published article and its Additional file 1.

## Abbreviations

SIRTs: Sirtuins
BC: Breast cancer
HR: Hazard ratio
CI: Confidence intervals
OS: Overall survival
DFS: Disease-free survival
TNM: Tumor-node-metastasis
IHC: Immunohistochemistry
qRT-PCR: Quantitative real-time polymerase chain reaction
HDAC: Histone deacetylases
MMP-2: Matrix metalloproteinase-2
EMT: Epithelial-mesenchymal transition
OXPHOS: Oxidative phosphorylation
HIF1α: Hypoxia-inducible factor-1a
SOD2: Superoxide dismutase 2
